# Effect of Zinc Added to Multivitamin Supplementation Containing Low-dose Vitamin A on Plasma Retinol Level in Children—A Double-blind Randomized, Controlled Trial

**Published:** 2007-03

**Authors:** Sunil Sazawal, Usha Dhingra, Saikat Deb, Maharaj K. Bhan, Venugopal P. Menon, Robert E. Black

**Affiliations:** ^1^ Department of International Health, Johns Hopkins Bloomberg School of Public Health, 615 North Wolfe Street, Room W5517, Baltimore, MD 21205, USA; ^2^ Centre for Micronutrient Research, Department of Biochemistry, Annamalai University, Annamalainagar 608 002, Tamilnadu, India; ^3^ Department of Pediatrics, All India Institute of Medical Sciences, New Delhi, India

**Keywords:** Vitamin A, Zinc, Zinc deficiency, Child, Retinol, Randomized controlled trials, Double-blind method, India

## Abstract

In a community-based double-blind randomized trial in children aged 6–35 months, both intervention and control groups received a multi-vitamin syrup containing vitamin A, while the intervention group had zinc gluconate (equivalent to 10 mg of elemental zinc) additional in the syrup. There was a significant decrease in diarrhoea and pneumonia in the intervention group. This study was undertaken to investigate if addition of zinc to vitamin A had improved plasma retinol levels, which, in turn, was responsible for the effects observed in the intervention group. In a randomly-selected subsample of 200 children—100 each from the intervention and the control group, plasma retinol levels after 120 days of supplementation were measured. There was no difference in the mean plasma retinol levels [the difference in the mean 0.46 μg/dL (95% confidence interval -1.42–2.36)] between the two groups following supplementation. No difference in plasma retinol levels was observed in the subgroups based on baseline nutritional status and plasma zinc levels. Addition of zinc to low-dose vitamin A in this study did not improve the vitamin A status of children and cannot explain morbidity effects of the intervention.

## INTRODUCTION

Vitamin A supplementation has shown a beneficial effect on childhood mortality (1), but its effect on morbidity is not clear. Deficiency of zinc (2) and vitamin A (3) are widespread in developing-country settings, and they often co-exist. Zinc deficiency could affect the bioavailability of vitamin A and, thereby, prevent the beneficial effect of vitamin A in reducing morbidity (4). In animal models, zinc deficiency is associated with reduced retinol levels. In zinc-deficient rats, zinc supplementation, not vitamin A supplementation, increased serum retinol levels (5). Data from human studies evaluating the effect of zinc supplementation on vitamin A status are limited and are inconclusive. Some studies have reported beneficial effects of zinc on plasma vitamin A concentration (6–8), while other studies failed to confirm these findings (9, 10).

In a double-blind randomized, controlled trial (11), we demonstrated the preventive effects of supplementation with zinc and vitamin A (intervention group) compared to the same preparation without zinc (control group). Supplementation reduced diarrhoea by 26% (13–38%) (11), persistent diarrhoea by 33% (6–52%) (12), and pneumonia by 45% (10–67%) (13). As the intervention group received both zinc and vitamin A, a possible hypothesis for this effect was that zinc supplementation improves the plasma retinol levels, which, in turn, is responsible for the preventive effects on morbidity. To evaluate this hypothesis, we undertook the present study in a random sample of 200 children.

## MATERIALS AND METHODS

### Main study

Details of the zinc supplementation trial within which the present study was nested have been reported earlier (11–13). Briefly, it was a randomized, controlled trial where both intervention and control groups received a daily dose of 5 mL of multi-vitamin syrup for 120 days. Each 5 mL contained vitamin A (240 μg of retinol equivalents), thiamine (0.6 mg), riboflavin (0.5 mg), vitamin B-6 (0.5 mg), cholecalciferol (2.5 μg) vitamin E (3 mg of α-tocopherol equivalents), and niacin (10 mg of niacin equivalents). The intervention group supplement, in addition to the above, also contained zinc gluconate equivalent to 10 mg of elemental zinc. Supplementation was doubled during diarrhoea to provide for additional losses. The supplements were formulated specially for the trial and masked for package, taste, and colour by Sandoz India Ltd., Mumbai, India.

A team of field assistants dispensed the assigned preparation to the child at home every day, except Sundays and holidays, when they left a measured dose in a separate vial for the mother to feed the child. Trained field workers visited each enrolled child at home once every five days, and information on compliance to intervention and morbidity for each of the previous five days was collected.

Venous blood was collected with monovette trace element-free heparinized syringes (Sarstedt, Newton, North Carolina, USA) for the estimation of plasma zinc concentrations at baseline and after 120 days of supplementation. Plasma zinc was analyzed using atomic absorption spectrometry (14) at the Department of Nutrition, University of Colorado, USA. The human research review committee at the All India Institute of Medical Sciences, the Johns Hopkins School of Hygiene and Public Health, and the World Health Organization approved the study.

### Vitamin A substudy

For the present study, a sample of 200 children (100 children aged less than 12 months and 100 children aged ≥12 months, 50 in each strata from the intervention and the control group) were randomly selected from 609 children in the main study. Originally, the sample size was determined to be sufficient for detecting a 15% increase with alpha of 0.05 and power of 90% within age-strata. Given observed results, the study had a power of 90% to detect 17% difference within two age-strata. For overall comparison, it has a power of 80% to detect 10% difference.

Of the 200 children selected, 184 children (90 in the intervention group and 94 in the control group) had plasma after 120 days of supplementation available. These samples were analyzed for plasma retinol at the John Hopkins nutrition laboratory using reverse phase, isocratic high-performance liquid chromatography.

Data were analyzed using the SPSS software for Windows (version 14; SPSS Inc., Chicago, USA). The statistics package of the National Center for Health Statistics (NCHS) (version 3; Centers for Disease Control and Prevention, Atlanta, Georgia, USA) was used for anthropometric calculation. Student's t-test, paired t-test, and chi-square were used for comparing the groups. Statistical significance was accepted at a probability level of 0.05. Exact methods were used for the calculation of confidence intervals using Stata (version 9.0).

## RESULTS

Of the 200 children selected for the study, 10 children in the intervention group and six in the control group did not consent to blood withdrawal; hence, the final analysis was done with 184 children. Children in the intervention and the control group were comparable for all assessed baseline characteristics, including plasma zinc concentrations (intervention 66.3±14.3 μg/dL; control 66.7±25.3 μg/dL) (Table 1). The characteristics of the children in this sample of 184 were also comparable with the overall sample of 609 in the main study (data not shown).

**Table 1.** Baseline comparison of children in the intervention and control groups

Feature	Vitamin A with zinc	Vitamin A without zinc
	(n=90)	(n=94)
Age (months) at enrollment*	14.8±8.0	15.2±8.5
5–11†	42 (46.7)	40 (42.6)
12–23†	32 (35.6)	35 (37.2)
24–36†	16 (17.8)	19 (20.2)
Nutritional status‡		
Normal†	34 (37.8)	40 (42.6)
Stunted†	39 (43.3)	30 (31.9)
Wasted†	8 (8.9)	8 (8.5)
Wasted and stunted†	9 (10.0)	16 (17.0)
Gender		
Males†	44 (48.9)	47 (50.0)
Plasma zinc levels in μg/dL at baseline*	66.3±14.3	66.7±25.3

*Mean+SD;

†Number (percentage);

‡Categorized as <-2 z-score using the National Center for Health Statistics standard

Plasma retinol concentrations after 120 days of the supplementation were very similar in the intervention and control groups (Table 2). Further, subgroup analysis by age (<12 months and ≥12 months), baseline plasma zinc levels (<60, 60–80, and >80 μg/dL), and nutritional status (normal, stunted, wasted, and wasted and stunted) did not show any difference in the plasma retinol levels between the two groups (Table 3). The proportion of children with post-supplementation plasma retinol below 20.0 μg/dL (vitamin A-deficient) was also not significantly different between the intervention (38%) and the control group (41%), odds ratio (OR)=1.17 (95% CI 0.62–2.20, p=0.61).

**Table 2.** Plasma retinol and zinc levels in the intervention and control groups after 120 days of supplementation

Variable	Vitamin A with zinc group			Vitamin A without zinc group			
	No.	Mean	Median (5, 95 percentile)	No.	Mean	Median (5, 95 percentile)	Difference in mean (95% CI)
Plasma retinol (μg/dL)	90	21.6±6.6	22.1 (10.1,32.8)	94	22.1±6.3	22.1 (10.9, 32.2)	-0.46 (-2.36,1.42)
Age <12 months*	46	20.4±6.8	20.6 (8.1,32.1)	48	22.2±6.9	21.9 (10.1, 32.8)	-1.77 (-4.62,1.06)
Age ≥12 months*	44	23.0±6.2	23.8 (11.5,32.8)	46	22.1±5.6	22.4 (11.9, 31.6)	0.90 (-1.59,3.40)
Plasma zinc (μg/dL)	90	86.3±35.7	73.5 (51.0,175.4)	94	66.3±25.0	62.0 (46.8, 97.0)	19.97 (11.03,28.91)

*Age at baseline;

CI=Confidence interval

**Table 3.** Difference in plasma retinol levels among both intervention and control groups after 120 days of supplementation in sub-groups of children by baseline plasma zinc and nutritional status

Veariable	Vitamin A with zinc group			Vitamin A without zinc group			Difference in mean (95% CI)
	No.	Mean	Median (5, 95 percentile)	No.	Mean	Median (5, 95 percentile)	
Baseline zinc levels							
<60.0 μg/dL	33	22.2±6.4	22.7 (10.6, 32.8)	36	23.1±6.1	22.7 (13.8,33.8)	-0.82 (-3.84,2.19)
≥60-<80 μg/dL	48	21.7±6.9	21.8 (10.3, 33.5)	50	21.5±6.3	21.8 (10.2,31.7)	0.14 (-2.50,2.80)
≥80.0 μg/dL	9	19.9±7.1	22.2 (7.9, 27.6)	8	22.2±7.5	21.5 (10.6,32.5)	-2.27 (-9.85,5.29)
Baseline nutritional status							
Normal	34	21.36±6.0	21.69 (10.8,29.97)	40	21.80±6.70	20.52 (10.6,33.8)	-0.44 (-3.41,2.53)
Stunted	39	21.20±7.0	21.13 (8.5, 32.93)	30	22.75±6.84	22.47 (9.2,32.5)	-1.54 (-4.92,1.83)
Wasted	8	23.27±6.0	22.19 (15.4, 35.1)	8	22.17±6.68	23.24 (13.5,31.5)	1.10 (-5.74,7.94)
Wasted and stunted	9	23.68±8.4	27.02 (5.8, 29.0)	16	21.95±4.12	23.05 (16.0,30.1)	1.72 (-3.44,6.89)

CI=Confidence interval

## DISCUSSION

In the present study, significant improvement in zinc status was not accompanied by differences in the post-supplementation plasma retinol levels between the children getting vitamin A with zinc and the children getting vitamin A without zinc. A potential limitation to the study is that interpretation of these results is based on two assumptions: that vitamin A status for the two groups was comparable at baseline and that serum retinol has adequate sensitivity to assess improvement in vitamin A status. The trial was a randomized double-blind trial, and in all assessed variables, the groups were comparable at baseline (plasma zinc and nutritional status). Serum retinol done immediately after the completion of 120 days of the supplementation is a reasonably-sensitive indicator of vitamin A status (15, 16). Thus, it is unlikely that these potential limitations would have biased the results.

Zinc is important for synthesis of retinol-binding protein (RBP), prealbumin, and hepatic cellular RBP (cRBP) and for the intracellular transport of retinol within liver hepatocytes. Vitamin A binds to RBP within the cytoplasm of the hepatocyte forming holo-RBP, which is secreted into the blood. Prealbumin forms a tri-molecular complex with retinol and RBP which conserves vitamin A by preventing its filtration and loss in the urine. Hence, zinc is essential for both intra- and inter-cellular transport of vitamin A (17, 18). Zinc also aids in the absorption of vitamin A in the intestine (19).

Our findings are consistent with findings of only two other studies in children, which had a longer duration of supplementation (20, 21). These studies did not find any difference in the post-supplementation vitamin A levels among the groups receiving zinc alone, vitamin A alone, vitamin A with zinc, and placebo group.

A recent randomized community-based trial in Bangladesh, with a factorial design of vitamin A and zinc, did not find any difference in the mean retinol levels between zinc or zinc/vitamin A groups compared to the placebo (8). In the same study, in the sub-group of children with vitamin A levels below 20.0 μg/dL at baseline, there was a significant reduction in the proportion of children with plasma retinol levels of <20.0 μg/dL after supplementation in the zinc and vitamin A group compared to the vitamin A-alone group. Children who were vitamin A-deficient at baseline in the Bangladesh study were deficient despite a mega dose of vitamin A given to them in the previous six months. In essence, children enrolled in that study represented a non-responding vitamin A-deficient subpopulation.

We also did not find any difference in the proportion of children with <20 μg/dL of plasma retinol levels between the two groups after supplementation. The number of children with vitamin A levels of <20 μg/dL in this study was not very different from Bangladesh (8). This would exclude differences due to lack of severe deficiency in the study population. All the three studies reporting an interaction (6–8) gave a large dose of vitamin A and had a short-term effect assessment (1–2 week(s)). Short-term high-dose supplementation of vitamin A may produce effects that are eventually counteracted by adaptation over a longer period of supplementation. Similar results have earlier been reported in trials in selected subpopulations, such as severely-malnourished children where supplementation with 40 mg/dL of zinc for 5–10 days resulted in a significant increase in vitamin A levels, but there was no change in vitamin A-deficient children (7). In our study, no difference was found in the plasma retinol levels in children who were zinc-deficient at baseline (plasma zinc of <60.0 μg/dL) or children who were below <-2 z -score of the NCHS standards for weight-for-height or age.

In conclusion, given similar plasma retinol levels in both intervention and control groups after 120 days of supplementation, it is unlikely that the observed effects of morbidity with zinc supplementation in our study (11–13) could have been mediated by an interaction of zinc with vitamin A. Further studies are needed to look into this question and to evaluate if giving a single large dose of vitamin A and zinc or a large dose of vitamin A with prolonged zinc supplementation may potentially have different effects on vitamin A status. As this has implications for supplementation programme, the effect of such a strategy needs to be evaluated in a community-based trial of adequate duration.

## ACKNOWLEDGEMENTS

The study was supported by the World Health Organization, Thrasher Research Fund, and United States Agency for International Development (Cooperative Agreement No. NIH.R29HD34724). The authors acknowledge gratefully the contributions of parents of participating children, Michael Hambidge, and Jaime Westcott for help with plasma zinc estimations, Keith P West for retinol estimation, and all the field staff of the project for their efforts and cooperation.
